# Design of composite adaptive controller with multilateral adaptive learning mechanism

**DOI:** 10.1038/s41598-025-26366-6

**Published:** 2025-11-26

**Authors:** Chao Niu, Yumei Yao, Zengliang Zhang, Mingxin Zuo

**Affiliations:** 1 School of Electrical Engineering, Henan Mechanical and Electrical Vocational College, Zhengzhou, 451191 China; 2 School of Mechanical and Electrical Engineering, Henan Mechanical and Electrical Vocational College, Zhengzhou, 451191 China; 3 Hebi Institute of Engineering and Technology, Henan Polytechnic University, Hebi, 458000 China

**Keywords:** Multilateral learning mechanism, Composite learning, Parameter uncertainty, Initial value assignment, Saturation transfer function, Engineering, Mathematics and computing

## Abstract

To enhance trajectory tracking performance for affine nonlinear systems with parametric uncertainties and improve parameter convergence under interval excitation, this paper proposes a multilateral cooperative adaptive learning mechanism. The initial parameter values are assigned based on available data distribution or predefined bounds when unknown. A composite learning adaptive controller estimates system uncertainties using multilateral learning outputs. Adaptive update laws for unknown parameters and multilateral weights are designed using parameter estimation errors and approximation errors, with a saturation function constraining weight variation rates to suppress oscillations. Experimental results on an inverted pendulum system demonstrate the superiority of the proposed controller over two conventional adaptive controllers.

## Introduction

Parameter uncertainty is a problem to be faced in controller design and has been widely concerned for a long time. In direct adaptive control, it is usually unnecessary to consider the convergence of unknown parameters, and more attention is paid to the control performance of system state tracking. In this mode, the adaptive rate of parameters is directly designed from the tracking error of the system, and then the adaptive controller is designed. In indirect adaptive control, it is not only concerned with the trajectory tracking of the system state, but also the convergence of unknown parameters. When the identified parameters are accurate enough, this method can compensate the uncertainty to a large extent and reduce the tracking error of the system. In the usual controller design, both methods are effective^1,2^. In order to achieve good trajectory tracking characteristics of the four-rotor, a robust controller based on adaptive dynamic programming was proposed in reference^3^, which can eliminate the adverse effects of parameter uncertainty and external disturbance on the system. There are also uncertainties of parameters in spacecraft, an online estimator is designed to identify unknown parameters^4^. Robot system and multi-robot system have become a research hotspot, in which the problem of parameter uncertainty cannot be ignored, the application of adaptive neural network compensation mechanism is a common solution^5–7^. Similarly, fuzzy approximators are also a more commonly used intelligent method for approximating uncertainty, which has been applied in references^8,9^. By designing an event triggering strategy and an adaptive sliding mode estimator, reference^10^ compensates for the effects of interference and parameter uncertainty. In high-speed train system and virtual coupled train system, adaptive non-singular terminal sliding mode controller and adaptive nonlinear controller are designed respectively, which can ensure good tracking and safety of the system^11,12^. The Hamilton scheme of adaptive error port control is proposed in reference^13^, and the robust adaptive control scheme is designed in reference^14,15^, both of which are used to solve the parameter uncertainty problem in the system. A time derivative enhanced parallel hard physics-informed neural networks (T-phPINN) was developed to adaptively solve non-Fourier heat conduction problems, achieving notably high accuracy^16^. To address challenges in compressible flows modeling for aerospace applications, CF-DeepONet was proposed in reference^17^, integrating branch network extracted features for rapid flow fields prediction. In addition, some studies have solved the uncertainty problem in complex nonlinear systems to some extent^18–21^. Although these works are validated, the convergence error of the parameters is not fully considered in the design of the adaptive rate, or the convergence of the parameters to the true value cannot be guaranteed when the persistent excitation (PE) condition is not satisfied. Because the strict PE condition is difficult to achieve in most physical systems, some researchers consider designing the adaptive rate under the interval excitation (IE) condition, in order to obtain the adaptive controller with good robustness and generalization.

Composite learning adaptive control is one such method, which can achieve accurate convergence of unknown parameters under IE conditions^22^. In the design of parameter adaptive rate, the method not only uses the instantaneous tracking error, but also constructs the prediction error to accurately identify the unknown system parameters. The following are some research advances on composite learning control methods in recent years. In order to achieve parameter convergence within a fixed time and improve the speed of parameter convergence, references^23–25^ proposed composite learning command filtering backstepping control, global composite learning timing control and fixed time adaptive neural network control, respectively. In the robot system, some scholars used the composite learning mechanism to design the adaptive update rate of the neural network^26,27^, and also designed the composite learning non-singular terminal sliding mode controller and the composite learning impedance controller^28,29^ to achieve the convergence of parameter estimation errors and impedance errors. In order to achieve good control characteristics of dynamically positioned ships and underactuated autonomous underwater vehicle, references^30,31^ designed a composite learning method to ensure an independent and flexible convergence trend of parameters. Reference^32^ considers a predicator-based composite learning technique to improve the ability of accurate estimation of unknown nonlinearities. In terms of prescribed performance control, references^33,34^ combined the use of composite learning control technology to ensure the stability of the system and the ability to approximate unknown nonlinearities. In affine nonlinear systems, based on the model reference adaptive control scheme, a composite learning adaptive controller was designed in^35^. Reference^36^ designed a composite learning controller based on the actor critic network and interference observer to achieve good trajectory tracking and vibration suppression capabilities. And references^37–39^ have designed effective control methods based on composite learning. From these studies, it can be seen that composite learning adaptive is a novel adaptive control scheme, which has a good performance in the problem of parameter uncertainty. However, the convergence speed and precision of the parameters depend on the control parameters of the design, and are more sensitive to the selection of parameters. When the control parameters are not sufficiently adjusted, the convergence process of unknown parameters may be slow. Therefore, it is necessary to design an adaptive adjustment mechanism with easy parameter adjustment, fast response speed and high convergence accuracy to further improve the adaptive control method of composite learning.

In order to solve the problem of slow convergence and strong dependence of unknown parameters in traditional adaptive control system of composite learning with parameter uncertainty, a new multilateral cooperative adaptive learning mechanism is proposed in this paper to improve the adaptive adjustment process of composite learning, and then a controller with higher convergence accuracy of unknown parameters and tracking errors is designed. Multilateral cooperative adaptive learning mechanism is a multi-branch adaptive iterative strategy based on the same structure. The adaptive structure of each branch is the same, and multiple uncertain approximation outputs are obtained by passing in different initial values of pre-assigned adaptive parameters. The uncertainty estimator in the control closed loop is obtained by the linear fusion of the multilateral weight and it is used as the adaptive term of the controller signal. In order to solve the adverse effects caused by the unknown initial value of adaptive parameters and the rapid change rate of multilateral weight parameters, the parameter initial value allocation strategy and saturation conversion function are further designed to improve the controller performance. The specific contributions are as follows

1. A multilateral cooperative adaptive learning mechanism was proposed and designed, and a new controller was obtained by integrating it with the traditional composite learning adaptive controller in reference^35^, which effectively improved the convergence speed and accuracy of unknown parameters and reduced the state tracking error. The design of the multilateral mechanism is based on the same adaptive structure, and it is not necessary to design each adaptive loop separately when applying, and multiple uncertainty approximation loops can realize adaptive synchronization update without adding additional burden on calculation.
2. Different from the unique initial parameter values set in reference^41^, this paper considers that different initial values have different effects on the control action, and designs the parameter initial value allocation strategy based on the potential possible laws of the data. By assigning the initial values of unknown parameters to multiple combinations in a unique way, different adaptive initial values of parameters are constructed for the multilateral. Through cyclic iteration and linear fusion of multilateral weight, the information output from different combinations can be fully utilized to quickly and accurately approximate the true values of parameters.
3. In order to constrain the change rate of multilateral weight parameters and prevent bad jitter in complex nonlinear objects, a saturation conversion function is designed to improve the design of multilateral cooperative adaptive learning mechanism. In general, the saturation conversion function has an adjustable boundary parameter, which is used to constrain the maximum value of the multilateral weight change rate. After the transformation of the nonlinear function, the change rate of the weight parameter can be stabilized in the bounded interval, which makes the action of the control system smoother.

The remaining parts are arranged as follows: In section 2, the parameter uncertainty problem and trajectory tracking control problem to be solved are proposed. In section 3, a combined feedforward feedback controller is designed under the framework of model reference adaptive adaptation, and the initial parameter assignment problem and corresponding solution are proposed. A multilateral composite learning adaptive controller is designed by integrating composite learning adaptive control and multilateral learning mechanism. In section 4, the Lyapunov stability proof is presented. In section 5, the inverted pendulum system is taken as the control object, and the simulation experiments of trajectory tracking and parameter convergence are completed compared with two adaptive controller. In section 6, the conclusion is given.

The symbols $\mathbb {R}$, $\mathbb {R}^{+}$, $\mathbb {R}^n$, $\mathbb {R}^{n\times n}$ represent real numbers, positive real numbers, *n*-dimensional real vectors, and $n\times n$-dimensional real matrices, respectively. $W^*\in \mathbb {R}^v$ represents the expected unknown parameter, $\hat{w}_i\in \mathbb {R}^m$ represents the vector composed of *m* estimated parameters of the *i*-th unknown parameter, $\bar{w}_j\in \mathbb {R}^v$ represents the *j*-th group of estimated parameters, where *v* represents the dimension of the unknown parameter, and *m* represents the number of sides of the unilateral. $|\cdot |$ represents the absolute value symbol, and $\Vert \cdot \Vert$ represents the Euclidean-norm of a vector. $sat(z,l_d)$ represents the saturation conversion function of the design, where *z* is the input variable, which can be either a scalar or a vector, and $l_d\in \mathbb {R}$ is the constraint boundary.

## Problem formulation

The effectiveness of the controller design based on model reference adaptive control has been widely verified. In the following, the control problem under the framework of model reference adaptive control is introduced by taking the single-input single-output affine nonlinear system as the control object. The reference system is a linear system, and the state space equation is

1 $$\begin{aligned} \dot{x}_r=A_rx_r+b_rr \end{aligned}$$

where, $x_r\in \mathbb {R}^n$ represents the state of the linear reference system, $A_r\in \mathbb {R}^{n\times n}$ is the system matrix, which is usually stable, $b_r=[0,\cdots ,0,1]^T$, and *r* is exogenous excitation signal, which can be arbitrarily set. In the actual industrial scene, it is often necessary to track a certain trajectory. In this case, *r* can be set as a matching signal, and under the action of the control signal, the system can track this trajectory.

Affine nonlinear system is a nonlinear system with a specific structure, and its state space equation can be described as

2 $$\begin{aligned} \dot{x}=\Lambda x+b\left( f\left( x\right) +u\right) \end{aligned}$$

where $x\in \mathbb {R}^n$ is the state of the affine nonlinear system, the model reference adaptive control is to ensure that the state of the nonlinear system can track the state of the linear reference system well. $\Lambda \in \mathbb {R}^{n\times n}$ is the system matrix of the known part of the model, which is a constant matrix, $b=[0,\cdots ,0,1]^T$, $f(x){:}\mathbb {R}^n\mapsto \mathbb {R}$ is the uncertain part of the model, and $u(t)\in \mathbb {R}$ is the control input.

Considering the parameter uncertainty problem in^35^, the uncertain part of the model can be parameterized linearly

3 $$\begin{aligned} f\left( x\right) =W^{*T}\Phi (x) \end{aligned}$$

The unknown parameter is represented as $W^*=\left[ w_1,w_2,\cdots ,w_v\right] ^T\in \mathbb {R}^v$, that is, there are *v* uncertain parameters, and correspondingly, $\Phi =[\phi _1,\phi _2,\cdots ,\phi _v]^T\in \mathbb {R}^v$ is the excitation vector, and all excitation functions are known.

### Remark 1

The parameter uncertainty problem considered in this paper refers to unknown parameters that can be expressed in the form of linear parameterization as shown in Equation (3). The excitation functions of the parameters are usually known to accurately identify the unknown parameters. Unknown parameters in nonlinear forms are not considered here.

The objective of the control is to make the state *x* of the affine nonlinear system track the state $x_r$ of the linear reference system, and to identify the true values of the unknown system parameters online. It is assumed that the states $x_r$ and *x* are measurable, the value of the state quantity can be obtained in real time, and $(\Lambda ,b)$ is controllable.

### Remark 2

In parameter uncertainty problems, only parameters are unknown, and the corresponding function vectors can be accurately represented by mathematical language. Usually, it is only necessary to pay attention to the convergence trend of parameters and the final convergence result. However, in some complex systems or scenarios, although the number of unknown parameters is limited, the number is large. At this time, it is troublesome to observe the changes of a large number of parameters at the same time, but substituting equation (3) can view the problem from the perspective of system uncertainty. Multiple parameters are fused together, and it is easy to observe the approximation degree of system uncertainty. It should still be noted that such treatment reduces the dimension of observation. In some cases, even if the unknown parameters do not converge, the system uncertainty still has a good approximation effect. Therefore, when observing the approximation degree of the system uncertainty, it is also necessary to pay attention to the convergence of some parameters to truly reflect the control effect.

### Definition 1

^40^ When there are constants $t_e,\tau _d,\sigma \in \mathbb {R}^+$ and $t_e>\tau _d$ makes $\int _{t_e-\tau _d}^{t_e}\Phi (\tau )\Phi ^T(\tau )d\tau \ge \sigma I$ true, the bounded signal $\Phi (t)\in \mathbb {R}^n$ is interval excitation (IE) on the interval $[t_e-\tau _d,t_e]$.

### Definition 2

^40^ A bounded signal $\Phi (t)\in \mathbb {R}^n$ satisfies the persistent excitation (PE) condition when there are constants $\sigma ,\tau _d\in \mathbb {R}^+$ makes $\int _{t-\tau _d}^t\Phi (\tau )\Phi ^T(\tau )d\tau \ge \sigma I,\forall t\ge 0$.

## Controller design

### Combined feedforward feedback controller

Define the tracking error signal as

4 $$\begin{aligned} e(t)=x_r\left( t\right) -x\left( t\right) \end{aligned}$$

Take the derivative of time and substitute it into equations (1) and (2) to get an expression for the differential error

5 $$\begin{aligned} \dot{e}=\Lambda e+\left( A_r-\Lambda \right) x_r+b_rr-b\left( f\left( x\right) +u\right) \end{aligned}$$

A new state vector $x_{re}=[{x_r}^T,r]^T$ is introduced, which is composed of the state of the linear reference system and the exogenous excitation signal, and is used to design the feedforward controller. In the framework of model reference adaptive, an adaptive controller containing feedforward feedback and adaptive terms is considered. The feedback control signal is based on the tracking error of the system, the feedforward control function is based on the new state vector $x_{re}$, and the adaptive term is designed as the output result of multilateral composite learning. Among them, multilateral composite learning is a strategy of weighted fusion of multiple composite learning adaptive processes, and learning through the deviation between each part and the system uncertainty reference value. The controller can be designed as^40^

6 $$\begin{aligned} u=k_e^Te+k_r^Tx_{re}-u_{ad} \end{aligned}$$

where, $k_r\in \mathbb {R}^{n+1}$ is the feedforward control gain, $k_e\in \mathbb {R}^n$ is the feedback control gain, and $u_{ad}$ is the adaptive compensation term.

For ease of analysis, the term containing $x_{re}$ is designed as

7 $$\begin{aligned} bk_r^Tx_{re}=\left( A_r-\Lambda \right) x_r+b_rr \end{aligned}$$

By substituting equation (6) and equation (7) into equation (5), the dynamic expression of error after reduction can be obtained as

8 $$\begin{aligned} \dot{e}=\Lambda e-b(f\left( x\right) +k_e^Te-u_{ad}) \end{aligned}$$

#### Remark 3

Regarding the selection of the control parameters $k_e$ and $k_r$, first of all, the selection of $k_r$ should satisfy the equation relationship in Equation (7). Then, it should be noted that the value of $k_e$ is conditional. That is, the result of the calculation of $\Lambda -b{k_e}^T$ should be strictly Hurwitz.

**Fig. 1:**
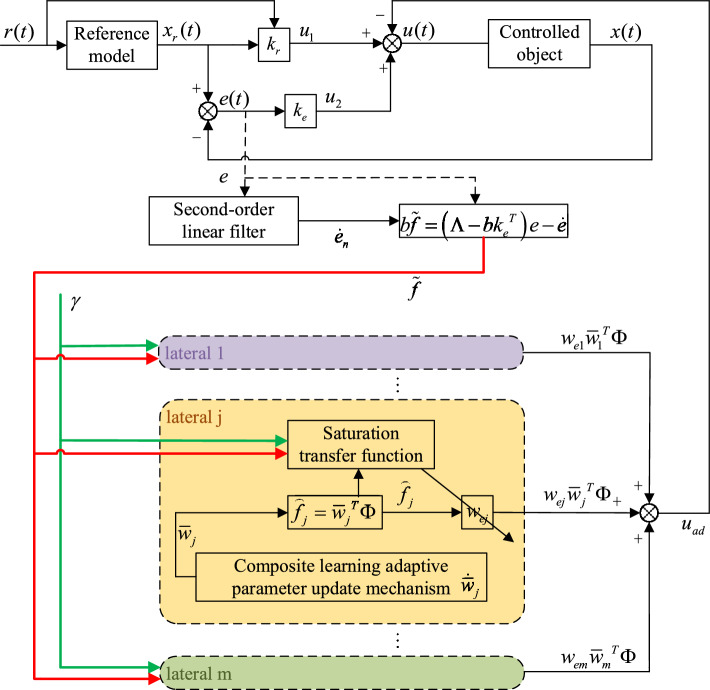
Model reference multilateral composite learning adaptive control structure.

As shown in Fig. 1, the input of the reference model is the external input reference signal *r*, which makes the state of the reference system change along with *r*. In general, *r* is set as the desired reference trajectory, the state change trend of the linear system is consistent with it, and the state of the nonlinear system gradually approaches the state of the linear system under the control. The reference signal and the linear reference state together become the feedforward action $u_1$ of the controller, and the tracking error is designed as the feedback control signal $u_2$, and the adaptive term $u_{ad}$ is subtracted from both to get the controller *u*. The tracking error *e* passes through the second-order linear filter to generate the differential error $\dot{e}_{n}$, and both of which flow into the multilateral composite learning mechanism, and the multilateral learning result is the third term of the controller.

#### Remark 4

The blue area in Fig. 1 is the designed multilateral composite learning adaptive mechanism, which is one of the innovation parts of this paper. It can be seen that the tracking error and its differential signal are used as the input of this part to calculate the approximation error $\tilde{f}$ of the system uncertainty. Then, the approximation error and adaptive learning rate gain $\gamma$ are fed into multiple adaptive adjustment channels with the same adaptive structure, and the output of each unilateral uncertainty approximation value weighted by multilateral weight $w_{ej}$ is obtained by the adder. To distinguish the unilateral with different adaptive parameters, the first, *j*-th, and last adaptive adjustment channels are represented by purple, orange, and green backgrounds, respectively. Among them, the adaptive channel with orange background is the most representative, completely drawing a single adaptive adjustment structure. The update output of the *j*-th estimation parameter $\bar{w}_j$ is completed by the composite learning adaptive parameter update mechanism, and then the corresponding system uncertainty estimate $\hat{f}_j$ is calculated directly. The *j*-th unilateral output is obtained by linear multiplication with the multilateral weight parameter $w_{ej}$. In addition, in order to update the multilateral weight parameter $w_{ej}$, the required variable combination is passed to the saturation conversion function, and the parameter adaptive update rate after constraints is output to avoid the jitter phenomenon caused by excessive parameter adjustment. Some of these symbols are not yet defined and will be explained one by one below.

### Parameter initial value assignment strategy for parameter uncertainty

In system control tasks with uncertain parameters, online identification of unknown parameters is a common approach. Each parameter is initially assigned a value and evolves during system operation to adapt to the control process. Under PE condition, unknown parameters may converge; otherwise, it’s difficult to achieve convergence with only instantaneous data correction. A composite learning adaptive control method combines direct and indirect adaptive control, builds prediction errors based on past data, and enables parameter convergence under IE condition with the joint action of instantaneous and historical data.

To enhance parameter convergence accuracy, a finite number of likely initial values for each unknown parameter are selected based on data characteristics and combined with other possible initial values to form multiple parameter estimators with different initial conditions. Initial value assignment has two modes: 1) Offline data exists to predetermine parameter data distribution; 2) Parameter initial value characteristics are unknown. For the first case, interval division is based on data distribution property, and boundary values are taken as initial values. The second case, more common in practice, assumes parameters follow a uniform distribution, determines possible upper and lower bounds in advance, and obtains initial values through uniform sampling. Mathematical language is used below to analyze parameter uncertainty with uniformly distributed data.

It is known that the system has *v* unknown parameter, that is, $w_i\left( i=1,2,\cdots ,v\right)$, which is the true value of the parameter, and $\hat{w}_{i}$ may be used to represent the corresponding estimate. Assuming *m* initial values are selected for each parameter, the multiple estimated parameters of the *i*-th parameter can be expressed as

9 $$\begin{aligned} \hat{w}_i=\begin{bmatrix}\hat{w}_{i1}&\hat{w}_{i2}&\cdots&\hat{w}_{im}\end{bmatrix}^T \end{aligned}$$

where $\hat{w}_{ij}$ represents the *j*-th estimate of the *i*-th parameter. $\tilde{w}_{ij}:=w_i-\hat{w}_{ij}$ is defined as the estimation error of the *j*-th estimate of the *i*-th parameter, which is later used in the design of the update rate of the parameter of the estimate.

#### Remark 5

Since the parameters are assumed to be uniformly distributed, and the initial value distribution results are obtained by sampling at equal intervals, $\hat{w}_{ij}^0$ is used to represent the initial value of the *j*-th th estimate of the *i*-th parameter, then multiple initial values meet the characteristics of monotone increasing or decreasing with respect to *j*, that is, ${\hat{w}_{ij}^0}>{\hat{w}_{ik}^0}\left( j>k\right)$ or ${\hat{w}_{ij}^0}<{\hat{w}_{ik}^0}\left( j<k\right)$, which monotonicity is arbitrary.

There are *v* unknown parameters. If all combinations of different parameters with different initial values are considered, the problem of computation explosion will be encountered when *v* is large, and more combination forms are easy to cause unnecessary trouble. In fact, this problem can be minimized, and the *m* as the total number of combinations can greatly simplify the calculation process. At this time, only the parameters with the same subscript *j* are taken as a combination to form a parameter estimator, and $\bar{w}_j\left( j=1,2,\cdots ,m\right) \in \mathbb {R}^v$ is used to represent such a combination of parameters, described in Table 1.

10 $$\begin{aligned} \bar{w}_j=\begin{bmatrix}\hat{w}_{1j}&\hat{w}_{2j}&\cdots&\hat{w}_{vj}\end{bmatrix}^T \end{aligned}$$

**Table 1:** Initial value assignment for multilateral estimated parameters.

Lateral index	The estimation vector of unknown parameters
1	$$\bar{w}_1=\begin{bmatrix}\hat{w}_{11}&\hat{w}_{21}&\cdots&\hat{w}_{v1}\end{bmatrix}^T$$
2	$$\bar{w}_2=\begin{bmatrix}\hat{w}_{12}&\hat{w}_{22}&\cdots&\hat{w}_{v2}\end{bmatrix}^T$$
3	$$\bar{w}_3=\begin{bmatrix}\hat{w}_{13}&\hat{w}_{23}&\cdots&\hat{w}_{v3}\end{bmatrix}^T$$
⋮	⋮
*j*	$$\bar{w}_j=\begin{bmatrix}\hat{w}_{1j}&\hat{w}_{2j}&\cdots&\hat{w}_{vj}\end{bmatrix}^T$$
⋮	⋮
*m*	$$\bar{w}_m=\begin{bmatrix}\hat{w}_{1m}&\hat{w}_{2m}&\cdots&\hat{w}_{vm}\end{bmatrix}^T$$

The error vector formed by the combination and the true value of the parameter is denoted as $\tilde{w}_j$, there is

11 $$\begin{aligned} \tilde{w}_j=W^*-\bar{w}_j=\begin{bmatrix}\tilde{w}_{1j}&\tilde{w}_{2j}&\cdots&\tilde{w}_{vj}\end{bmatrix}^T \end{aligned}$$

The closer the initial parameter estimation range is to the actual initial parameter value, the better the transient behavior of the system in the control process. Assuming that the predetermined upper and lower bounds of the *i*-th parameter are $l_{ai},l_{bi}(l_{ai}<l_{bi})$, and the length of the range is expressed by $s_i:=l_{bi}-l_{ai}$, the initial value $\hat{w}_{ij}^0$ can be calculated as

12 $$\begin{aligned} \hat{w}_{ij}^0=l_{ai}+\frac{(l_{bi}-l_{ai})(j-1)}{m-1}=l_{ai}+\frac{s_i(j-1)}{m-1} \end{aligned}$$

Given that the excitation vector is $\mathrm {\Phi }$, the estimated value of the system uncertainty calculated by different parameter combinations can be expressed as

13 $$\begin{aligned} \hat{f}_j=\bar{w}_j^T\Phi \end{aligned}$$

where, $\hat{f}_j$ represents the estimated value of the system uncertainty calculated from the combination of the *j*-th parameter, and the deviation from the true value *f* is expressed as $\tilde{f}_j$.

Consider introducing a multilateral learning weight $w_{ej}\left( j=1,2,\cdots ,m\right)$, representing the weight of the *j*-th system uncertainty estimate, to calculate the final system uncertainty estimate $u_{ad}$. Instead of using a single uncertainty estimate directly, multiple uncertainty estimates are relearned in the form of weighting. On the one hand, the robustness of the system is improved, and on the other hand, the output result of the uncertainty estimator is more reliable.

14 $$\begin{aligned} u_{ad}=\sum _{j=1}^mw_{ej}\hat{f}_j \end{aligned}$$

Similarly, the initial value of the multilateral learning weight is also to be considered, and it is useful to use $w_{ej}^0$ to represent the initial value of the *j*-th multilateral weight. In a simple example, several weight initialization methods such as random initial value, equal interval initial value and normalized average initial value are tested. The results show that normalized average initial value is the best one of them. The multilateral weights can then be initialized to

15 $$\begin{aligned} {w_{ej}^0}=\frac{1}{m} \end{aligned}$$

#### Remark 6

Regarding the selection of the initial values $l_{ai}$ and $l_{bi}$ of the parameters, it can be based on the law of the prior distribution, or the parameters can be assumed to be unknown, so that the initial values are set within a reasonable range. Usually, when the possible range of the unknown parameters is not known, it is assumed that the upper and lower bounds of the parameters are values symmetric about the origin, and a small positive integer is selected in the positive direction. In addition, the multilateral parameter *m* should be chosen as an integer as small as possible, as long as it can ensure that the system is within the allowable error range. Because as *m* increases, some unilateral terms contribute little to the final formed uncertainty approximation value and occupy a certain amount of computational resources. Even if this part of the resources is acceptable, the entire controller can still be optimized by reducing unnecessary unilateral terms.

### Composite learning adaptive mechanism

The composite learning adaptive control strategy uses both instantaneous data and historical data. In the design process, not only tracking error is considered, but also prediction error is constructed, so that the tracking error can still converge to zero and the unknown parameter converges to the true value when the PE condition is not satisfied. Ideally, the method can get the actual value of the unknown parameter in a finite time, but the model in the actual scene is complex, the existence of noise and interference signals may prolong the identification process or the parameter convergence to the neighborhood near the true value, and the identified unknown parameter still has a small deviation. In order to improve the accuracy of identification, a multilateral learning mechanism is adopted to deal with this challenge. For the detailed design process, see the relevant content of multilateral composite learning. In the initial parameter assignment, $\bar{w}_j$ is used to represent the *j*-th parameter combination. The composite learning adaptive mechanism of the parameter combination is described below.

The symbol of the tracking error is known as *e*, a new prediction error $\varepsilon :=\Theta _e\tilde{w}_j$ is defined, and

16 $$\begin{aligned} \Theta _e:&= {\left\{ \begin{array}{ll}\Theta (t),t<T_e\\ \Theta (T_e),t\ge T_e& \end{array}\right. } \end{aligned}$$

17 $$\begin{aligned} \Theta (t):&= \int _{t-\tau _d}^t\Phi (x(\tau ))\Phi ^T(x(\tau ))d\tau \end{aligned}$$

where $\tau _d\in \mathbb {R}^+$ is an integral period and $T_e\ge \tau _d$ is a specific time involved in the calculation. In the general composite learning adaptive controller design^35^, $T_e$ is the time when the minimum singular value of $\Theta (t)$ reaches the maximum, here, as with most controller parameters, it is considered as an artificial parameter.

Here we need to solve the calculation problem of prediction error $\varepsilon$, which is not difficult to get from the definition of $\tilde{w}_{j}$ in equation (11)

18 $$\begin{aligned} \Theta (t)\tilde{w}_j(t)=\Theta (t)W^*-\Theta (t)\bar{w}_j(t) \end{aligned}$$

Since $\Phi (x)$ is known and $\bar{w}_j$ is available in the control process, the second term on the right side of the equation can be computed. The more difficult thing to deal with is the first term on the right of the equation, which is ${\Theta }W^{*}$. Here is a solution. Rewrite equation (8) as follows

$$\begin{aligned} \dot{e}=\Lambda e-b\left( f\left( x\right) +k_e^Te-u_{ad}\right) \end{aligned}$$

Since the approximation value $\hat{f}_j$ to the uncertainty of the *j*-th parameter combination is itself similar result to $u_{ad}$, the variable is replaced here

19 $$\begin{aligned} \begin{aligned} \dot{e}&=\Lambda e-b\left( f+k_e^Te-\tilde{f}_j\right) \\&=\left( \Lambda -bk_e^T\right) e-b\tilde{f}_j \end{aligned} \end{aligned}$$

Multiply both sides by $\Phi (x(t))$, integrate over the interval $\left[ t-\tau _d,t\right]$, transform some terms and get

20 $$\begin{aligned} \Theta \left( t\right) W^*=\int _{t-\tau _d}^t-\Phi \left( x\right) \left( \dot{e}_n-\bar{w}_j^T\Phi -bAe\right) d\tau \end{aligned}$$

In equation (21), it is still necessary to obtain the differential signal $$\begin{array}{c}\dot{e}_n\end{array}$$ of the tracking error *e*. In order to reduce the complexity of the calculation, this variable is obtained by using a second-order linear filter. The model of the filter is

21 $$\begin{aligned} {\left\{ \begin{array}{ll} \dot{\hat{e}}_n=\hat{e}_{n+1}\\ \dot{\hat{e}}_{n+1}=-2\zeta \omega \hat{e}_{n+1}+\omega ^2\left( e_n-\hat{e}_n\right) \end{array}\right. } \end{aligned}$$

where

22 $$\begin{aligned} {\left\{ \begin{array}{ll} \hat{e}_n(0)=e_n(0)\\ \hat{e}_{n+1}(0)=0& \end{array}\right. } \end{aligned}$$

Filter variable $\hat{e}_n$ is the corresponding part of signal $e_n$, filter variable $\hat{e}_{n+1}$ is the corresponding part of signal $\dot{e}_n$, and parameters $\omega \in \mathbb {R}^+$ and $\zeta \in \mathbb {R}^+$ represent the natural frequency and damping ratio respectively. Among them, the natural frequency and damping ratio are controllable parameters of the second-order linear filter. In order to meet the suitable filtering form, these two variables should adapt to the changing trajectory of the filtering state with time, so as to obtain more accurate filtering output results. The state differentiation of the tracking error is obtained by using the filter, which avoids the complexity of the analytical calculation and reduces the computational burden of the control process. In addition, in the process of second-order linear filtering, the adverse effects of high frequency noise such as measurement noise on the system will be reduced.

The adaptive control rate of composite learning is

23 $$\begin{aligned} \dot{\bar{w}}_j=\gamma _ee^TPb\Phi (x)+\gamma _ek_w\varepsilon \end{aligned}$$

where, $\gamma _e\in \mathbb {R}$ is the learning rate, *P* is the positive definite matrix designed later, and $k_w\in \mathbb {R}$ is the predicted gain. The parameter adaptive update rate is composed of two parts. The first item makes use of the tracking error of the system, so that the parameter adjustment has the ability to quickly track the reference trajectory; the second item makes use of the prediction error constructed earlier, so that the historical data can be fully used in the parameter adjustment, and the parameter convergence can still be completed under the condition of IE.

**Fig. 2:**
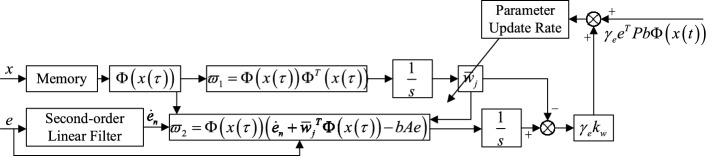
Composite learning adaptive parameter $\bar{w}_{j}$ update mechanism.

As shown in Fig. 2, the relevant historical data of state *x* is recorded in the memory, $\tau$ represents the past time, $\varpi _1$ can be calculated from the past excitation vector $\Phi (x(\tau ))$, and then the integration output is obtained through the integrator. The tracking error *e* passes through the second-order linear filter to get the error differential $\dot{e}_{n}$, and the two are involved in the calculation of $\varpi _2$ together with the historical data $\Phi (x(\tau ))$ and the current parameter vector $\bar{w}_{j}$, and then the integrated output is obtained through the integrator. The newly constructed prediction error $\varepsilon$ is obtained by subtracting the dot product of the $\varpi _1$ integral result from $\varpi _2$ and the parameter vector $\bar{w}_{j}$. The product of the prediction error and the learning gain is the second term of the parameter update rate, plus the first term calculated by the tracking error *e*, to form the update formula of the parameter vector $\bar{w}_{j}$.

### Multilateral composite learning adaptive mechanism

Based on the previous composite learning adaptive mechanism, the design of the multilateral composite learning adaptive mechanism is completed. The detailed process is as follows. The forward process and initial value of the multilateral learning mechanism are known from equations (14) and (15), where $\hat{f}_j$ is the fitting result of the system uncertainty obtained from the transformation of parameter uncertainty in the adaptive process of composite learning. Note the difference between the adaptive term $u_{ad}$ and the true uncertainty *f* as $\tilde{f}$, then

24 $$\begin{aligned} \tilde{f}=f-u_{ad} \end{aligned}$$

Substitute into equation (8) to get

25 $$\begin{aligned} \dot{e}=\left( \Lambda -bk_e^T\right) e-b\tilde{f} \end{aligned}$$

Due to the existence of unknown parameters, the actual uncertainty *f* cannot be directly calculated and $\tilde{f}$ is difficult to determine. Look carefully at the above formula, transposition can be obtained

26 $$\begin{aligned} b\tilde{f}=\left( \Lambda -bk_e^T\right) e-\dot{e} \end{aligned}$$

As previously known, the differential of the tracking error *e* can be obtained by a second-order linear filter, so equation (26) is solvable. The adaptive rate of multilateral learning weight $w_{ej}$ is

27 $$\begin{aligned} \dot{w}_{ej}=\gamma \hat{f}_j\tilde{f} \end{aligned}$$

where, $\gamma \in \mathbb {R}$ is the learning rate. The adaptive rate makes use of the deviation between the adaptive term and the actual uncertainty, and can adjust the multilateral weight to adapt to the actual uncertainty, which is advantageous for approximating the real value of the unknown parameter to a certain extent.

In order to prevent excessive oscillation caused by too fast adjustment of the adaptive rate, it is necessary to use some means to reduce the peak value of the adaptive rate. In the design of adaptive controller, there are some methods, such as $\sigma$ correction, *e* correction, projection mechanism, which can limit the range of adaptive rate adjustment. In this paper, the saturation conversion function is used to achieve this effect. A new saturation conversion function is designed as

28 $$\begin{aligned} sat\left( z,l_d\right) =\frac{l_d}{1+e^{-z}}-\frac{l_d}{1+e^z} \end{aligned}$$

where *z* is the input of the nonlinear function, and $l_d\in \mathbb {R}^+$ is the constraint boundary, limiting the input to the interval $[-l_d,l_d]$. Then equation (27) can be further improved to equation (29).

29 $$\begin{aligned} \dot{w}_{ej}=sat\left( \gamma \hat{f}_j\tilde{f},l_d\right) \end{aligned}$$

After constant updating, $w_{ej}$ will constantly adapt to the real uncertainty and may eventually converge to a definite value. Each parameter combination forms a composite learning adaptive process, and the obtained uncertainty estimate $\hat{f}_j$ is multiplied by *m* multilateral weights to obtain the final adaptive term, which constitutes a complete multilateral composite learning adaptive controller.

The parameter selection process for the controller design is outlined as follows:

**Step 1**: Based on the nature of linear parameterization, determine the dimension and boundary range of the parameters to be estimated. Assign the extreme values of the parameter range to the boundary parameters $l_{ai}$ and $l_{bi}$, where $l_{ai}$ typically represents the minimum value and $l_{bi}$ the maximum value. Considering both the multilateral principle and computational load, determine the dimension parameter *m* for the multilateral structure, partition the range for each unknown parameter accordingly, and initialize the estimated parameters $\bar{w}_j$ in a predefined form.

**Step 2**: Different initialization methods for the multilateral weight parameters $w_{ej}$ can be investigated. Through comparative analysis, an optimal initialization rule can be identified. In this study, normalized equal values are chosen as the initial values for the multilateral weight parameters. This approach normalizes the magnitudes across different branches, preventing abrupt signal variations during composite computation, while equal initial weighting ensures that all branches are treated equally at the start before entering their respective adaptive phases.

**Step 3**: Each branch of the multilateral structure incorporates a second-order linear filter. The filter parameters $\zeta$ (damping ratio) and $\omega$ (natural frequency) are often determined empirically, ensuring that the filtered output adapts appropriately to the controller’s adaptive behavior.

**Step 4**: For gain and learning rate parameters such as $\gamma$, $\gamma _e$, and $k_w$, a sensitivity analysis or trial-and-error method is used to identify their adjustable ranges. An iterative tuning process-gradually increasing or decreasing the values is then applied, combined with observation of the system’s response characteristics, to determine the optimal settings. Additionally, the boundary parameter $l_d$ must be tuned by considering both the amplitude variations of signals within the system and the magnitude of the learning rate gains, so as to ensure robust stability throughout the system’s operation.

#### Remark 7

As can be seen from equation (28), the saturation conversion function is a continuous nonlinear function, and $l_d$is an amplification factor used to appropriately adjust the upper and lower bounds of the nonlinear function curve. At the same time, $l_d$also defines the variable interval of the adaptive rate of the multilateral weight parameter, so that the adaptive rate signal is always bounded. The function is monotonically increasing and does not change the positivity of the incoming variable, and the output value is the ordinate of the corresponding point of the incoming variable on the nonlinear curve. Without the saturation conversion function, the adaptive update rate may be a relatively large value, which will affect the adaptive parameters will have a large increment, resulting in overshoot phenomenon, resulting in a certain degree of jitter in the internal state of the system. However, under the constraint of the saturated transfer function, the adaptive update rate is always within the defined constraint range, which effectively weakens the jitter phenomenon, and the saturated transfer function is a continuous curve, which is conducive to the smooth adjustment of parameters.

## Stability analysis

### Theorem 1

Considering the model reference adaptive control problem of affine nonlinear systems (2) with parameter uncertainty, in order to ensure that state *x* can track state $x_r$ of linear reference systems (1) well and that unknown parameters can converge to their true values under IE conditions, the controller (6), the update rate of composite learning parameters (23) and the update rate of multilateral weights (29) are designed. If the choice of gain parameter $k_r$ satisfies equation (7) and $k_e$ satisfies the condition that $\Lambda -b{k_e}^T$ is a strict Hurwitz matrix, then the closed-loop system can achieve global asymptotic stability, and the closed-loop signal is bounded, and the tracking error can converge to a small neighborhood of zero and the parameters converge.

### Proof

Define the multilateral weight vector $$W_m=\begin{bmatrix}w_{e1},w_{e2},\cdots ,w_{em}\end{bmatrix}^T$$, assume that there is a corresponding true value $W_m^*=\left[ w_{e1}^*,w_{e2}^*,\cdots ,w_{em}^*\right] ^T$, let $\tilde{W}_m$ represent the error vector between the multilateral weight vector and the true value, then

30 $$\begin{aligned} \tilde{W}_m=W_m^*-W_m \end{aligned}$$

For equation (25), matrix $A:=\Lambda -bk_e^T$ is introduced, then $k_e$ should be taken so that matrix *A* is strictly Hurwitz matrix. In this case, the error dynamic is written as

31 $$\begin{aligned} \dot{e}=Ae-b\tilde{f} \end{aligned}$$

If matrix $Q=Q^{T}>0$ is chosen, there is a unique solution $P=P^T>0$ to the Lyapunov equation.

32 $$\begin{aligned} A^TP+PA=-Q \end{aligned}$$

Define the Lyapunov energy function as

33 $$\begin{aligned} V=\frac{1}{2}e^TPe+\frac{1}{2\gamma _e}\sum _{j=1}^m\tilde{w}_j^T\tilde{w}_j+\frac{1}{2\gamma }\tilde{W}_m^T\tilde{W}_m \end{aligned}$$

There are three items on the right side of the equation, which are tracking error term, composite learning adaptive error term and multilateral learning weight error term. For individual analyses, label them $V_1$, $V_2$, and $V_3$ in order from left to right.

34 $$\begin{aligned} V_1&= \frac{1}{2}e^TPe \end{aligned}$$

35 $$\begin{aligned} V_2&= \frac{1}{2\gamma _e}\sum _{j=1}^m\tilde{w}_j^T\tilde{w}_j \end{aligned}$$

36 $$\begin{aligned} V_3&= \frac{1}{2\gamma }\tilde{W}_m^T\tilde{W}_m \end{aligned}$$

Equation (34) is obtained with respect to time differentiation

37 $$\begin{aligned} \begin{aligned} \dot{V}_{1}&=\frac{1}{2}\dot{e}^TPe+\frac{1}{2}e^TP\dot{e}\\&=-\frac{1}{2}e^TQe-e^TPb\tilde{f} \end{aligned}\end{aligned}$$

Similarly, derivation of $V_2$ is obtained by substituting the composite learning adaptive rate formula (23), combining the definition of uncertainty approximation error $\tilde{f}_j$ and prediction error $\varepsilon$ get

38 $$\begin{aligned} \begin{aligned} \dot{V}_{2}&=-\frac{1}{\gamma _e}\sum _{j=1}^m\tilde{w}_j^T\dot{\bar{w}}_j \\&=-\sum _{j=1}^m\tilde{w}_j^T\left( e^TPb\Phi +k_w\varepsilon \right) \\&=-e^TPb\sum _{j=1}^m\tilde{f}_j-\sum _{j=1}^m\tilde{w}_j^T\Theta _e\tilde{w}_j \end{aligned}\end{aligned}$$

From the definition and calculation of ${\Theta }_{e}$, we can see that $\Theta _e\ge 0$ is always true and can be represented by its eigenvalue in $t\ge T_e$. Therefore, the second term in $\dot{V}_2$ is never greater than zero. The first item on it will be discussed at the end.

Then, the derivation analysis of $V_3$ is carried out, and the adaptive rate of multilateral weights is substituted, taking equation (27) as an example.

39 $$\begin{aligned} \begin{aligned} \dot{V}_{3}&=\frac{1}{\gamma }\tilde{W}_m^T\dot{\tilde{W}}_m \\&=-\frac{1}{\gamma }\sum _{j=1}^m\tilde{w}_{ej}\dot{w}_{ej} \\&=-\sum _{j=1}^m\tilde{w}_{ej}\hat{f}_j\tilde{f} \end{aligned}\end{aligned}$$

It’s easy to know, $\tilde{f}=\sum _{j=1}^m\tilde{w}_{ej}\hat{f}_j$, then equation (39) can be further written as

40 $$\begin{aligned} \begin{aligned} \dot{V}_{3}&=-\sum _{j=1}^m\sum _{k=1}^m\tilde{w}_{ej}\tilde{w}_{ek}\hat{f}_k\hat{f}_j\\&=-\tilde{f}^T\tilde{f}\le 0 \end{aligned}\end{aligned}$$

It is obtained by combining formula (37), formula (38) and formula (40)

41 $$\begin{aligned} \dot{V}=-\frac{1}{2}e^TQe-\sum _{j=1}^m\tilde{w}_j^T\Theta _e\tilde{w}_j-\tilde{f}^T\tilde{f}-e^TPb\sum _{j=1}^m\left( \tilde{w}_{ej}+1\right) \tilde{f}_j \end{aligned}$$

The first three numbers on the right side of the middle sign of equation (41) are non-positive numbers, and only the positive or negative of the fourth term remains to be discussed. It is not difficult to know that under normal circumstances, the above signals are bounded. Suppose that the error $\tilde{w}_{ej}$ of the multilateral weight satisfies condition $\left| \tilde{w}_{ej}\right| \le \bar{w}_{ej},\forall t\ge 0$, where $\bar{w}_{ej}$ is the constrained boundary of the error. $\lambda _{\max }(\cdot )$ is used to represent the maximum eigenvalue of the matrix, $\left\| \cdot \right\|$ is used to represent the norm of the vector, and Young’s inequality is applied to the fourth term, then

42 $$\begin{aligned} \dot{V}\le -\frac{1}{2}e^TQe-\sum _{j=1}^m\tilde{w}_j^T\Theta _e\tilde{w}_j-\tilde{f}^T\tilde{f}+C \end{aligned}$$

where

43 $$\begin{aligned} C=\frac{1}{2}m\lambda _{\max }\left( P\right) \Vert e\Vert +\frac{1}{2}\sum _{j=1}^m\left( \bar{w}_{ej}+1\right) ^2\tilde{f}_j^2 \end{aligned}$$

Even if such a positive number *C* exists, the system is still stable enough to converge to a certain range. When the control parameters are selected appropriately, $\dot{V}\le 0$ is constant, and the system meets the global asymptotic stability. In the special case, $\dot{V}$ may appear greater than zero in the initial adjustment phase, but as the first three items in equation (42) are always less than or equal to zero, some parameters converge to a sufficiently small range that $\dot{V}$ is ultimately no greater than zero. Based on the above analysis, the system has a certain stability, which can ensure that the nonlinear system can track the state of the linear reference system well, the parameters are bounded and convergent, and theorem 1 is proved. $\square$

## Simulation

In order to verify the effectiveness of the designed model reference multilateral composite learning adaptive controller, the inverted pendulum system was simulated in the MATLAB2016b software platform, and the simulation duration was set at 30s.

Linear reference model is set to

$$\begin{aligned} \dot{x}_r=\begin{bmatrix}0& 1\\ -1& -2\end{bmatrix}x_r+\begin{bmatrix}0\\ 1\end{bmatrix}r \end{aligned}$$

There are two states, set the initial value to $$x_r(0)=\begin{bmatrix}1,1\end{bmatrix}^T$$, the exogenous excitation input to $r=1\left( 10\le t<15\right)$, $r=-1.5\left( 20<t<25\right)$, otherwise $r=0$.

The affine nonlinear model of inverted pendulum system is

$$\begin{aligned} \dot{x}=\begin{bmatrix}0& 1\\ 0& 0\end{bmatrix}x+\begin{bmatrix}0\\ 1\end{bmatrix}(f+u) \end{aligned}$$

The initial state value is set to $x(0)=[1,1]^T$, the expected parameter is $W^*=[1,-1,0.5]^T$, and the excitation function vector is $\Phi \left( x\right) =\left[ e^{x_1x_2},\sin \left( x_1\right) ,x_2\left| x_2\right| \right] ^T$.

The multilateral composite learning adaptive controller, the composite learning adaptive controller (COM) in^35^ and the adaptive finite time command filtered backstepping controller (AFTCFBC) in^41^ are compared in the experiment. Let $m=7$ (M7-COM). The control gains $k_e$ and $k_r$ are set to $k_e=[50,30]^T$ and $k_r=[-1,-2,1]^T$ respectively. In equation (21), the parameters of the second-order linear filter are set to $\omega =100$, $\zeta =0.7$. $Q=10I$ in equation (32), where *I* is the identity matrix. Set the predetermined upper and lower bounds of all unknown parameters equal, that is, $l_{ai}=l_{ak}=-1(i\ne k)$, $l_{bi}=l_{bk}=1(i\ne k)$. The composite learning time parameter $T_{e}=5$, the value of delay time is equal to $T_e$, that is, $\tau _d=5$. Learning rate $\gamma _{e}=\gamma =5$, prediction gain $k_w=1$. The constraint boundary of the saturation conversion function is $l_d=2$. For fair comparison, the relevant parameters of the composite learning adaptive controller have the same values as those of the multilateral composite learning adaptive controller.

Specifically, the structure of AFTCFBC is expressed as

$$\begin{aligned} u=r-k_{e2}e_2-W_a^{T}\Phi _{a}+\dot{x}_{2c}-e_1-c_2v_2^{2\beta -1} \end{aligned}$$

$$\begin{aligned} \dot{W}_a=\gamma _a(\Phi _a(\dot{e}_2+e_1+k_{e2}e_2+c_2v_2^{2\beta -1})-\sigma _aW_a) \end{aligned}$$

where, $k_{e2}$ denotes the second element of the feedback gain vector $k_e$. The parameters $W_a$ and $\Phi _a$ represent the estimated parameter uncertainty and the excitation function utilized within the controller, respectively. The virtual control input $x_{2c}$ is defined as $x_{2c}=x_2+e_2$. The term $v_2$ corresponds to the compensated tracking error, which is designed to mitigate tracking deviations through feedback correction. The parameters $c_2=0.1$ and $\beta =0.7$ are tuning parameters. Additionally, $\gamma _a=5$ specifies the adaptive learning rate for parameter adaptation, while $\sigma _a=10^{-4}$ is the correction gain parameter.

The integral of the absolute value of the tracking error is denoted as $J_x$, which is a performance indicator of the tracking error, and the integral of the absolute value of the fitting error of the system uncertainty transformed from the parameter uncertainty is denoted as $J_f$, as a performance indicator of the prediction error. The tracking error performance indicators of system state 1 and state 2 are represented as $J_{x_1}$ and $J_{x_2}$ respectively. Furthermore, it is known that $J_x$ represents the error performance over the entire simulation period, reflecting the combined effects of transient and steady-state characteristics. To comprehensively evaluate the actual performance of the controller, the steady-state accuracy must also be considered. This is quantified by the performance index $J_x^-$, which denotes the system’s performance after the unknown parameters have been fully learned. The key distinction lies in the error accumulation period for $J_x^-$, which begins at $T_e$ (as opposed to the full simulation period for $J_x$).

$$\begin{aligned} J_x = \int _0^T|e|dt \end{aligned}$$

$$\begin{aligned} J_x^- = \int _{T_e}^T|e|dt \end{aligned}$$

$$\begin{aligned} J_f = \int _0^T\left| \tilde{\vartheta }\right| dt\left( \tilde{\vartheta }=\tilde{f},\tilde{f}_j\right) \end{aligned}$$

The simulation duration is 30 seconds, and the error performance indexes of the whole process are recorded in Tables 2 and 3.

**Table 2:** Comparison of error performance indexes of the three control methods.

	COM	AFTCFBC	M7-COM
$J_{x_1}$	0.63288	**0.16128**	0.26474
$J_{x_2}$	1.6062	1.264	**0.6537**
$J_{x_1}^-$	0.078616	0.095065	**0.0020011**
$J_{x_2}^-$	0.055701	0.14484	**0.0037014**
$J_f$	54.2812	458.0228	**21.2952**

As can be seen from Table 2, the performance indexes of M7-COM’s tracking error and prediction error are superior to those of COM’s control method. Among them, the performance indexes of M7-COM’s state-1 tracking error are about 41.8% of those of COM’s method, and that of M7-COM’s state-2 tracking error are about 40.7% of those of COM’s method. The prediction error performance index of M7-COM is about 39.2% that of COM method. Compared to AFTCFBC, the proposed control method M7-COM achieves optimal performance across all metrics except for $J_{x_1}$. As evidenced by the data in the last three rows of the table, AFTCFBC exhibits the worst performance across these metrics, indicating its inferior capability in approximating unknown parameters compared to composite learning-based controllers. Overall, while AFTCFBC employs certain strategies to enable rapid state tracking of the reference trajectory, its relatively weak learning ability necessitates local readjustments when encountering trajectory variations or disturbances, requiring adaptation to new control conditions. In summary, the proposed M7-COM controller demonstrates superior numerical performance.

**Table 3:** Prediction error performance indexes of multiple composite learning processes.

Index *j*	$J_f$
1	64.6429
2	51.8073
3	44.0419
4	41.8478
5	40.6509
6	44.509
7	59.4054

In Table 3, the cumulative errors of seven composite learning adaptive processes are listed. Compared with 21.2952 of M7-COM method in Table 2, the prediction error performance indicators of the composite learning adaptive process are all too large, indicating the effectiveness of the multilateral composite learning adaptive strategy. The distribution of multilateral initial values is obtained uniformly in the interval $[-1,1]$. In Table 3, the composite learning adaptive process of different initial values shows different error performance. As the index increases, the performance index data first becomes smaller and then becomes larger. In the initial value state of index 5, the multilateral minimum value appears.

**Fig. 3:**
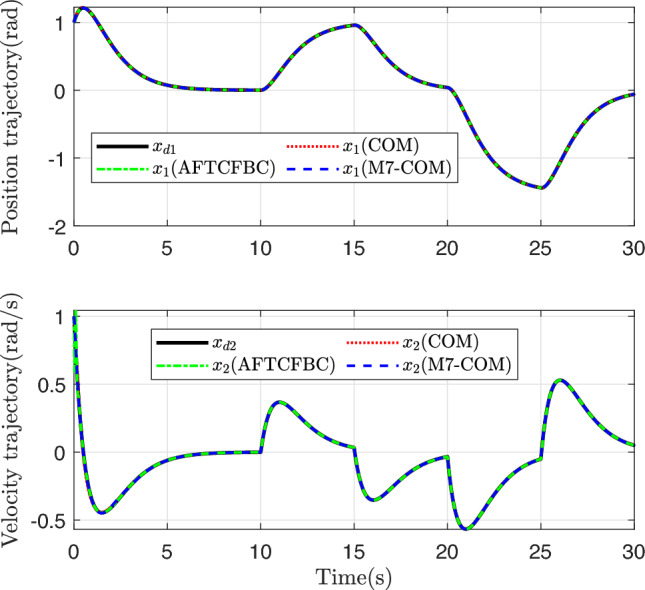
System state tracking curve.

**Fig. 4:**
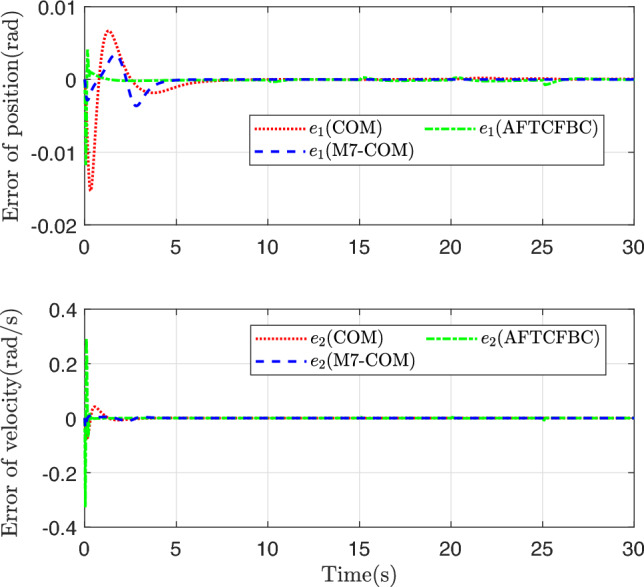
System state tracking error.

**Fig. 5:**
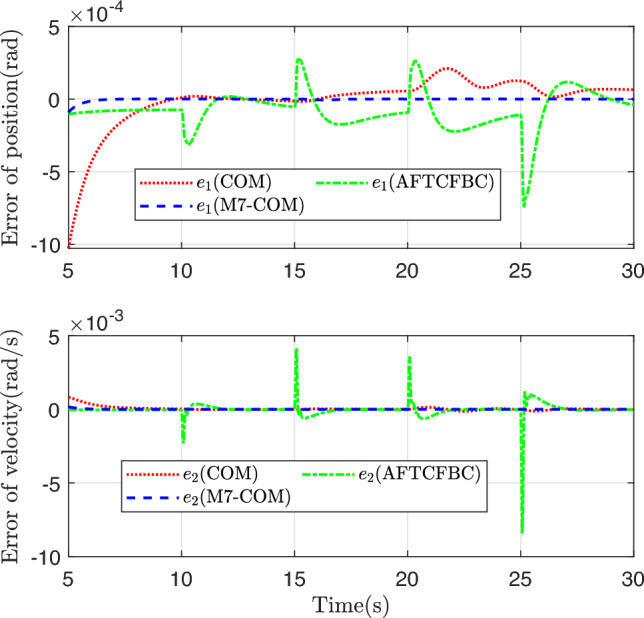
System state tracking error after 5s.

**Fig. 6:**
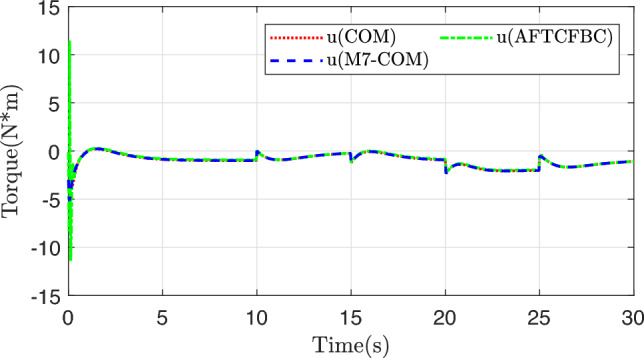
Control input signal.

Figure 3 shows the trajectory curves of the two states of the system tracking the linear reference system state. Both methods achieve good tracking, and it is difficult to distinguish between them from the whole. The state tracking error of the system is plotted in Figs. 4 and 5, and there are obvious differences. Comparison with COM Method, the M7-COM method shows better transient performance, which is specifically reflected in smaller overshoot and shorter adjustment time, and the small neighborhood that has been approaching zero at the moment of 5s. The difference between the two is that the M7-COM control method has higher accuracy and stronger adaptability. As can be seen from Fig. 4, the AFTCFBC method converges the fastest, but there is a large amplitude of tracking error during the initial adjustment phase. Observing Fig. 5, it is evident that the composite learning-based method exhibits strong robustness in the later stages, particularly the proposed M7-COM method, while AFTCFBC shows significant fluctuations when faced with high-order discontinuous reference trajectory. It should be noted that, in order to effectively estimate the unknown parameters in the system, composite learning-based control methods require error data of sufficient intensity in the early stages to construct the parameter adaptive update rate. Therefore, although the tracking error of M7-COM is greater than that of AFTCFBC during the transient phase, it maintains a sufficient excitation level to enable the rapid and accurate identification of unknown parameters, which is more conducive to dealing with high-order discontinuous reference trajectories. It can be observed from Fig. 6 that the control signals of the three control methods have similar amplitudes and variation trends.

**Fig. 7:**
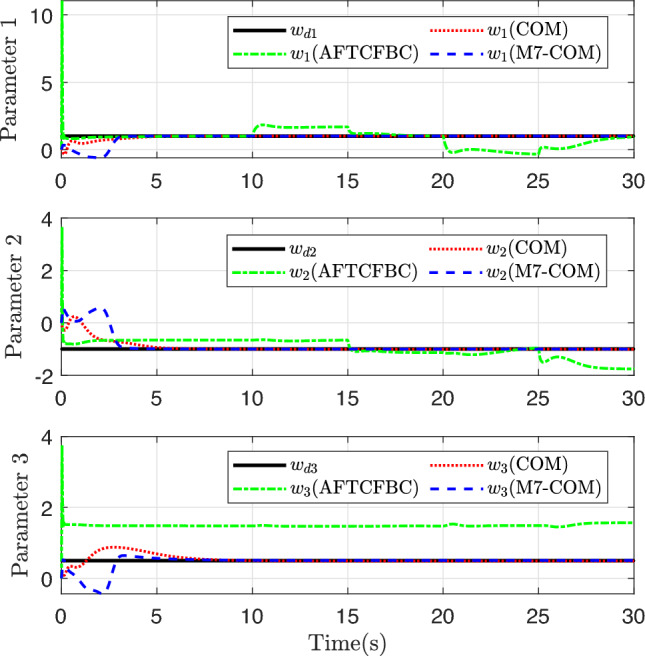
Convergence of the unknown parameters.

Figure 7 reflects the parameter convergence of the three control methods. The black line represents the expected parameter values. The red dotted line, the green dot-dashed line, and the blue dashed line represent the parameter convergence trajectory of COM, AFTCFBC, and M7-COM, respectively. From the convergence states of the three parameters, the convergence accuracy of the M7-COM control method is highest, and the coincidence degree with the expected line is higher in the later stage. From the initial stage, the parameter error of M7-COM is greater than that of COM method at 0 3 s, but the principle of adaptive control by composite learning is not difficult to imagine, and the process generates a large amount of historical data, without a certain incentive strength, it is difficult to ensure that the parameter convergence to the desired value. Therefore, it can be seen that the M7-COM method stores a strong excitation level in this time period. At about 3 s, the parameters converge sharply to the true value, and the convergence speed and intensity are much greater than that of the COM control method. It can be seen from the figure that COM method is inferior to M7-COM control method in both the speed and accuracy of parameter convergence. Unlike composite learning-based methods, the green trajectory line of AFTCFBC consistently fails to align with the desired black line. This is because the controller’s parameter adaptive update rate relies solely on instantaneous data without leveraging historical information, preventing the excitation matrix from achieving full rank. It is evident that during the adaptive updates of Parameter 1 and Parameter 2, AFTCFBC approaches the reference line for a certain period; however, the adaptive update of Parameter 3 consistently deviates from the expected value line, an issue that is inevitable.

**Fig. 8:**
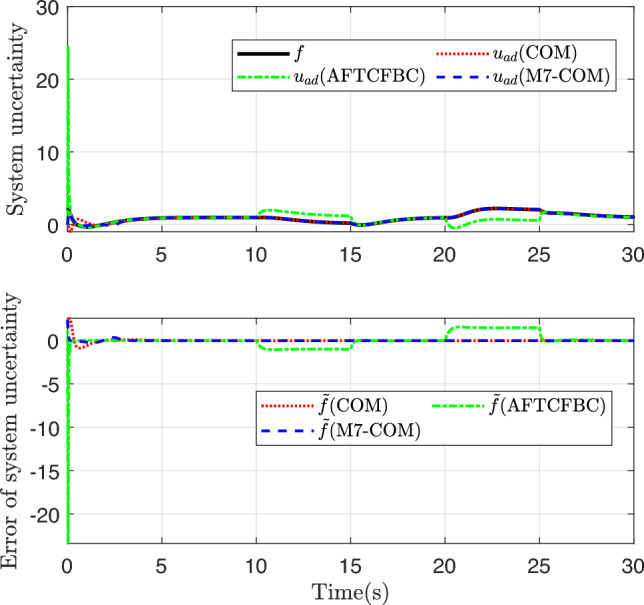
Fitting effect of system uncertainty.

To a certain extent, the ability to approximate parameter uncertainty can also be seen through the transformed system uncertainty approximation curve, which has certain advantages when there are many unknown parameters. Figure 8 shows the fitting curves and fitting errors of the three methods for the transformed system uncertainty. On the whole, the M7-COM control method has better fitting effect and is closer to the real system uncertainty at the initial stage, and the fitting error quickly converges to near zero. Recalling the parameter convergence curves in Fig. 7, it is found that the parameter convergence error of the M7-COM control method is relatively large from 0 to 3 s, but the fitting error of the system uncertainty is relatively small in Fig. 8. It is not difficult to know that the M7-COM control method not only guarantees the incentive strength of historical stored data in the initial stage, but also realizes the optimal compensation for the overall uncertainty of the system. In the figure, as the reference trajectory undergoes abrupt changes, AFTCFBC deviates significantly from the actual uncertainties twice, once after 10 seconds and again after 15 seconds, which highlighting the inferior performance of this method compared to the M7-COM approach.

**Fig. 9:**
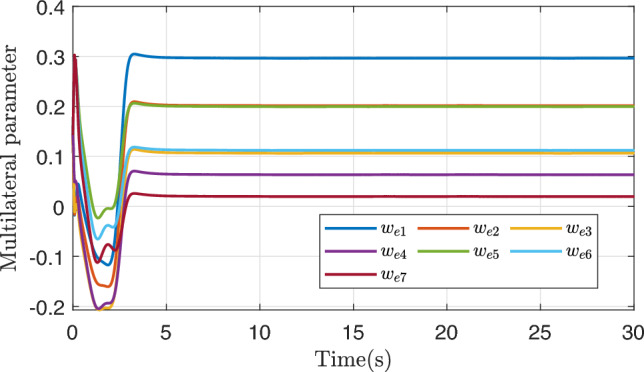
Learning change curve of each weight of the multilateral composite learning adaptive mechanism.

Figure 9 shows the change trajectory of multilateral weights. The initial values of 7 weights are all around 0.143. Through adaptive learning, each weight is dispersed and finally converges to a certain value. These curves have a similar trend of change, and all tend to converge to the truth value at about 3s. Even if the reference trajectory still has a jump at the later moment, they do not show obvious waves, showing strong robustness. From the numerical point of view, all the 7 curves converge to a positive value greater than zero, and the largest weight converges near 0.3, indicating that the 7 parameter combinations of composite learning adaptive have a positive effect on the multilateral learning result. According to the phenomenon of multilateral weight stratification, different parameter combinations correspond to different weights and have different contribution degrees to the final adaptive term.

**Fig. 10:**
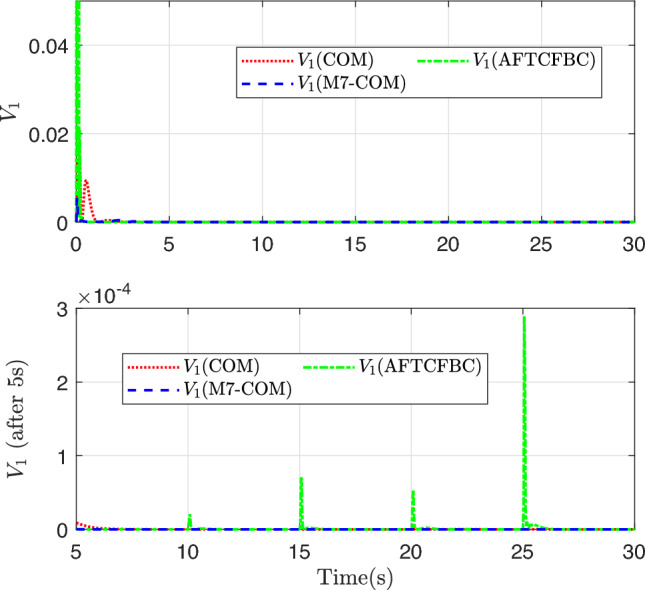
Stability convergence curve.

It can be seen from Fig. 10 that the variation curves of the Lyapunov energy function with respect to the tracking error for the two methods. Similarly, the red dotted line, the green dot-dashed line, and the blue dashed line represent COM, AFTCFBC, and M7-COM respectively. It can be observed that the line of M7-COM converges fastest and has smallest fluctuations in the initial stage. By observing the bottom subgraph, it can be seen that the AFTCFBC exhibits sharp spikes at the positions where the reference trajectory jumps. It is most sensitive to the sudden changes in the reference trajectory, which can be disadvantageous in certain scenarios.

**Fig. 11:**
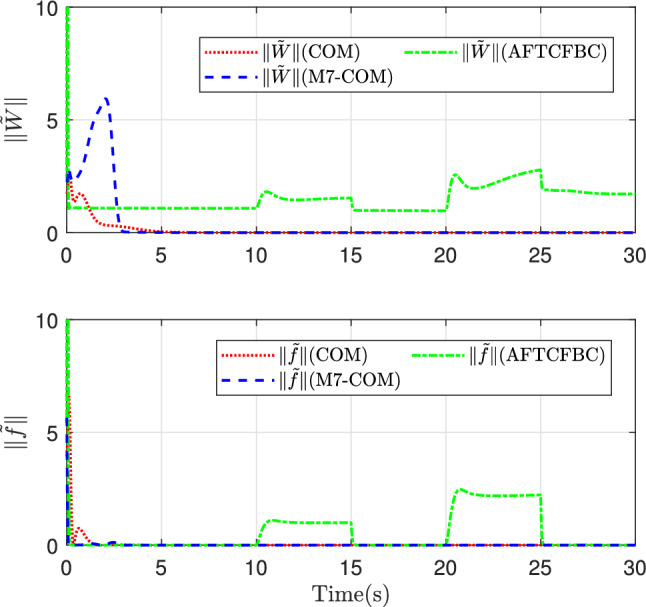
The norm of parameter error and approximation error.

In Fig. 11, the variation curves of the error norm of the estimated parameters in the three control methods and the approximation error norm curves of the transformed system uncertainty are depicted. Consistent with the previous figures, in the norm curve of the parameter estimation error, the value of the M7-COM method is relatively large in the initial stage, but it quickly drops to zero around 3 s, and then the norm of the parameter error remains almost continuously smallest. In terms of the approximation error norm of the system uncertainty, the M7-COM method has the best approximation effect, remaining at relatively small values throughout the process, which shows its superiority over the other two methods.

According to the above analysis, compared with the ordinary composite learning adaptive controller and the adaptive finite time command filtered backstepping controller, the multilateral composite learning adaptive controller not only guarantees the good tracking ability of the nonlinear system state, but also realizes the convergence of unknown parameters faster and more accurately, and has a better performance in the proposed performance index.

## Conclusion

This paper addresses the trajectory tracking control and parameter convergence issues of affine nonlinear systems with parameter uncertainty. A multilateral composite learning adaptive controller is proposed based on the model reference adaptive control framework and composite learning adaptive principle, and verified in an inverted pendulum system. A combined feedforward-feedback controller is designed with adaptive term to compensate for the impact of uncertainties on trajectory tracking. An initial value assignment strategy for unknown parameters is presented, considering two distribution modes based on whether some initial values are known or completely unknown. Parameter update rates for both the composite learning adaptive loop and multilateral weights are designed, and a saturation conversion function is introduced to prevent rapid parameter changes from causing oscillations. The stability of the whole multilateral composite learning adaptive controller is analyzed using Lyapunov functions. In a trajectory tracking simulation experiment with an interval excitation reference trajectory on the inverted pendulum system, compared with the conventional composite learning adaptive controller and the adaptive finite time command filtered backstepping controller, the proposed controller shows the best performance in tracking error and prediction error performance indices. The multilateral composite learning adaptive control strategy offers a new controller design pattern and a solution to parameter convergence problems.

The designed multilateral composite learning adaptive controller is based on the theory of linear parameterization of system uncertainties. This approach is applicable to many nonlinear systems, such as robotics, mechanical systems, and aircraft, where unknown parameters—including mass, inertia, and friction coefficients—can be linearly parameterized. However, linear parameterization often relies on approximations, and the system inherently retains nonlinear characteristics. Using nonlinear parameterization to describe the system can better model real-world dynamics, thereby yielding improved response outcomes. To extend this method to nonlinearly parameterized systems, two potential approaches are proposed. First, nonlinear neural networks can be employed to approximate arbitrary continuous uncertainties. The controller structure can be modified into a nonlinear form, and the multilateral mechanism can be utilized to configure diverse combinations, resulting in a highly robust nonlinear approximator. Second, integration with fuzzy logic systems can be explored, where adaptive fuzzy systems serve as nonlinear approximators and are combined with the multilateral learning framework. In both cases, new challenges arise: the introduction of nonlinear approximators complicates the Lyapunov stability proof, requiring more detailed analysis, and the conditions for parameter convergence may become more stringent, necessitating guaranteed uniform ultimate boundedness of the approximation error for the overall uncertainty.

Although the proposed controller demonstrates superior performance in simulations, this study still has certain limitations. The integration of the multilateral learning mechanism with the composite learning method introduces relatively high computational complexity, which affects the real-time execution efficiency of the controller. On embedded platforms with limited computational resources, the real-time performance of the controller requires further consideration. In the future, we plan to pursue lightweight improvements to the controller by optimizing its structure, so as to enhance the efficiency of parallel computing and increase the controller’s response speed. Additionally, an event-triggered parameter update strategy could be designed to reduce unnecessary computations during system response. Finally, to further extend the proposed controller, integration with state observers could be considered to handle control scenarios where system states are not directly measurable.

## Data Availability

The datasets used during the current study available from the corresponding author on reasonable request.
